# Measuring the stereogenic remoteness in non-central chirality: a stereocontrol connectivity index for asymmetric reactions

**DOI:** 10.3762/bjoc.21.155

**Published:** 2025-09-30

**Authors:** Ivan Keng Wee On, Yu Kun Choo, Sambhav Baid, Ye Zhu

**Affiliations:** 1 Department of Chemistry, Faculty of Science, National University of Singapore, 3 Science Drive 2, Singapore 117543

**Keywords:** asymmetric reactions, axial chirality, catalysis, planar chirality, stereocontrol

## Abstract

Despite the rapid development of asymmetric synthesis, judging the remoteness of stereocontrol has remained an intuitive and empirical practice, particularly for reactions that create non-central chirality. We put forward a stereocontrol connectivity index to parameterize asymmetric reactions according to the bond connectivity relationships between the prochiral stereogenic elements, the reactive sites, and the stereochemical-defining substituents. The indices can be generated based on analysis of the chemical structures of the starting materials and products, without mechanistic insights of the transformation. Representative examples of reactions that establish point chirality, axial chirality, planar chirality, and “inherent chirality” are illustrated using the stereocontrol connectivity index produced following a unified 3-step process. Application of such stereochemical classification could facilitate the development of new synthetic methodologies and catalyst systems to construct diverse chiral molecules.

## Introduction

**Scheme 1:**
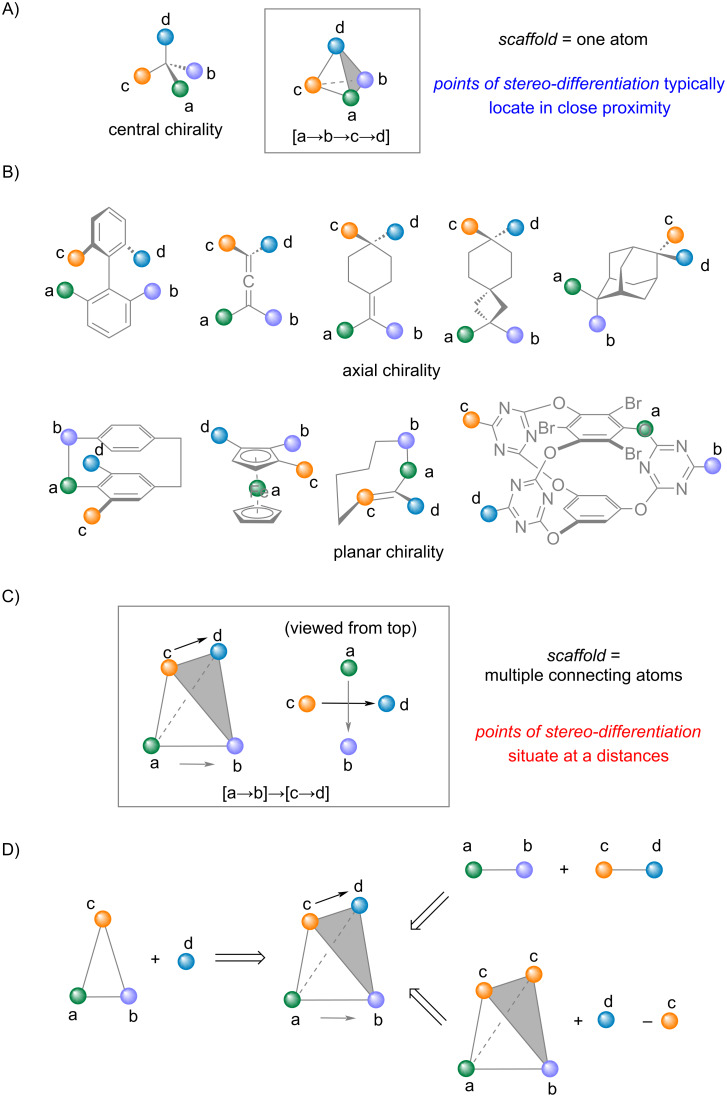
Illustration of chirality and the intrinsic remoteness of stereogenic elements for axial chirality and planar chirality.

Chirality is a ubiquitous and fundamental phenomenon in nature and thus holds an irreplaceable position in organic synthesis. At its most rudimental definition, chirality in a molecule is characterized by the absence of mirror planes and centers of inversion. Central chirality arises when four distinct substituents (a, b, c, and d) are arranged tetrahedrally around a central atom (Scheme 1A). Non-central chirality – such as axial and planar chirality – are becoming increasingly important in pharmaceuticals, catalysts, and advanced materials due to their unique stereogenic scaffolds and associated properties. Consequently, synthetic chemists have been pursuing molecules featuring these forms of non-central chirality, where the stereogenic elements are not localized on a single central atom (Scheme 1B).

While chemists often classify chirality (stereogenicity) into distinct types according to the stereogenic elements, such as central (point) chirality, axial chirality, and planar chirality, these categories are inherently related. They can be viewed as arrangements of groups within three-dimensional molecular frameworks that restrict conformational freedom. The geometric scaffolds can be a central atom, an axis, or a plane, and the combination and spatial arrangement of chemically distinct substituents establish the stereogenicity. Therefore, despite the apparent differences among these scaffolds, the various types of chirality are permutations of substituents on diverse stereogenic elements (Scheme 1C).

Although this analysis offers a unified view of chirality, the realization of these chiral systems through asymmetric synthesis is far from trivial. Stereogenic compounds can be generated from achiral starting substrates by various means that assemble the four distinct substituents (a, b, c, and d) (Scheme 1D). In the case of central chirality, the differentiating substituents are often directly attached to the newly formed stereocenter. By contrast, for non-central chirality, the pairs of substituents (a and b, c and d) are separated in space because the stereogenic scaffolds span multiple atoms. Consequently, bond cleavage and formation occur at positions that are distant from the stereogenic elements and remote from the actual points of differentiation among the substituents.

Intuitively, the intrinsic spatial separation among prochiral stereogenic elements, the reactive sites, and the stereochemical-defining substituents makes stereoinduction for non-central chirality using a chiral catalyst or reagent particularly challenging. However, a quantitative parameterization of asymmetric reactions remains unavailable, and the remoteness of stereocontrol for reactions that establish non-central chirality is judged based on empirical chemical intuition. While developing catalytic methods to establish remote stereogenic elements, we became increasingly interested in parameterizing the relay of stereochemical information from the chiral catalysts to the prochiral substrates. In this study, we propose a stereocontrol connectivity index that quantitatively characterizes asymmetric reactions. The index could serve as a basis for classifying asymmetric reactions according to the positioning of stereochemically relevant elements, independent of the type of transformation. Additionally, the index enables the identification of the minimal set of structural features in a molecule that are recognized by chiral catalysts to achieve stereocontrol.

## Results and Discussion

We envisaged that the stereocontrol connectivity index should reflect the bond connectivity of prochiral stereogenic elements, the reactive sites, and the stereochemical-defining substituents. These structural elements contribute to the transmission of chirality from the chiral catalysts and reagents to the prochiral substrates, thereby representing the minimal structural features recognized by chiral catalysts or reagents.

Furthermore, the index should be derived from straightforward analysis of the chemical structures of the substrates and the products without the need for conformational analysis and mechanistic understanding of the catalytic process. The chemical reaction is the movements of electrons that change the bond connections, which can be denoted by the bond break and bond formation. The stereochemical outcome is expressed as the chirality of the product, which can be designated following the Cahn–Ingold–Prelog rules.

We now put forward a stereocontrol connectivity index [i*_j_*] for a transformation that encodes the bond-connecting relationship between the establishment of chirality and the site of reaction. The index [i*_j_*] of asymmetric reactions can be assigned following a 3-step process:

**Step 1:** Identification of atoms involved in bond changes.

1.1 Determine which bonds are newly formed and which are cleaved in the transformation.

1.2 Label all atoms directly involved in these bond changes.

**Step 2:** Identification of atoms responsible for stereochemical outcome.

2.1 Identify the new stereogenic element formed in the product.

2.2 Determine, according to the Cahn–Ingold–Prelog (CIP) rules, the atoms that distinguish the newly created stereogenic element's configuration. These are the atoms whose identities represent the first point of difference between the set of substituents in assigning the configuration of the new stereogenic element. One or two sets of atoms could be identified.

a) These atoms labeled in step 1 are considered higher in priority than any other atoms.

b) If the comparison is down to between an atom already labeled in step 1 and one other atom, such comparison and this set of atoms are excluded. In other words, the number set of atoms will be reduced by one.

c) Consider "dummy" atoms from multiple bonds as lower priority than real atoms of the same type.

**Step 3:** Determination of stereocontrol pathways.

For each set of atoms identified in step 2:

3.1 Find the shortest path connecting the labeled atoms from step 1 to each atom identified in step 2.

3.2 Count the minimum number of connecting bonds (i).

3.3 Count the number of bonds shared (j) between the shortest paths.

The reaction is then assigned a stereocontrol connectivity index of [i*_j_*] or [i*_j_*,i’*_j’_*], depending on the number of sets of atoms identified in step 2 (one or two sets).

**Scheme 2:**
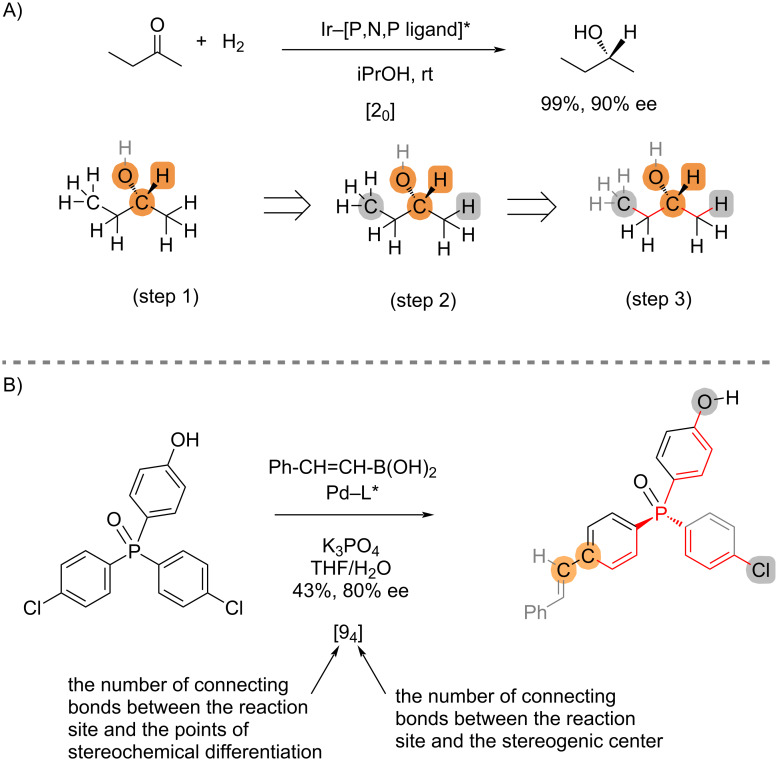
Illustrations of assignment using point chirality.

Before discussing applications to non-central chirality, we analyze two examples of stereoselective reactions that establish central chirality to illustrate the concept of stereogenic remoteness measured using the stereocontrol connectivity index (Scheme 2). A detailed process for assigning the index is shown in Scheme 2A for asymmetric hydrogenation of 2-butanone [1]. The atoms involved in bond cleavage and bond formation are highlighted in orange color. The atoms responsible for assignment of the stereochemical configuration of the products are highlighted in grey color. The shortest connecting bonds between them are colored red. Accordingly, the asymmetric hydrogenation of 2-butanone is designated as [2_0_] process because there are two connecting bonds between the stereogenic carbon and the stereochemical differentiation atoms, and they do not share a common path.

Typically, in stereoselective reactions involving central chirality, the proximity between the site of reaction and the points of stereo-differentiation means that the indices [i*_j_*] are expected to have small values. In general, *j* = 0 in the index [i*_j_*] of an addition reaction to two prochiral faces of a planar substrate, where the prostereogenic carbon is part of the reaction site. However, this is not always the case, particularly in remote desymmetrization reactions [2–8]. For instance, the catalytic desymmetrization of phosphine oxides [9] will be defined as a [9_4_] process (Scheme 2B). As such, the index [i*_j_*] indicates the number of connecting bonds (i) between the reaction site and the points of stereochemical differentiation, and the number of connecting bonds (j) between the reaction site and the stereogenic center. The indices are not defined by the reaction types, the mode of catalysis, or the nature of the stereogenic centers.

Unlike central chirality, the stereogenic remoteness of non-central chirality could not be measured using a central stereogenic atom as the starting point. The stereocontrol connectivity index allows parameterization for reactions that establish axially chirality and planar chirality regardless of the absence of stereogenic centers. Applications to reactions that forge axial chirality follow the same 3-step procedure. The two substituents at each end of the stereogenic axis are ranked based on the CIP priority rules, and the set(s) that do not involve bond formation and bond cleavage are used to identify the points of stereochemical differentiation.

**Scheme 3:**
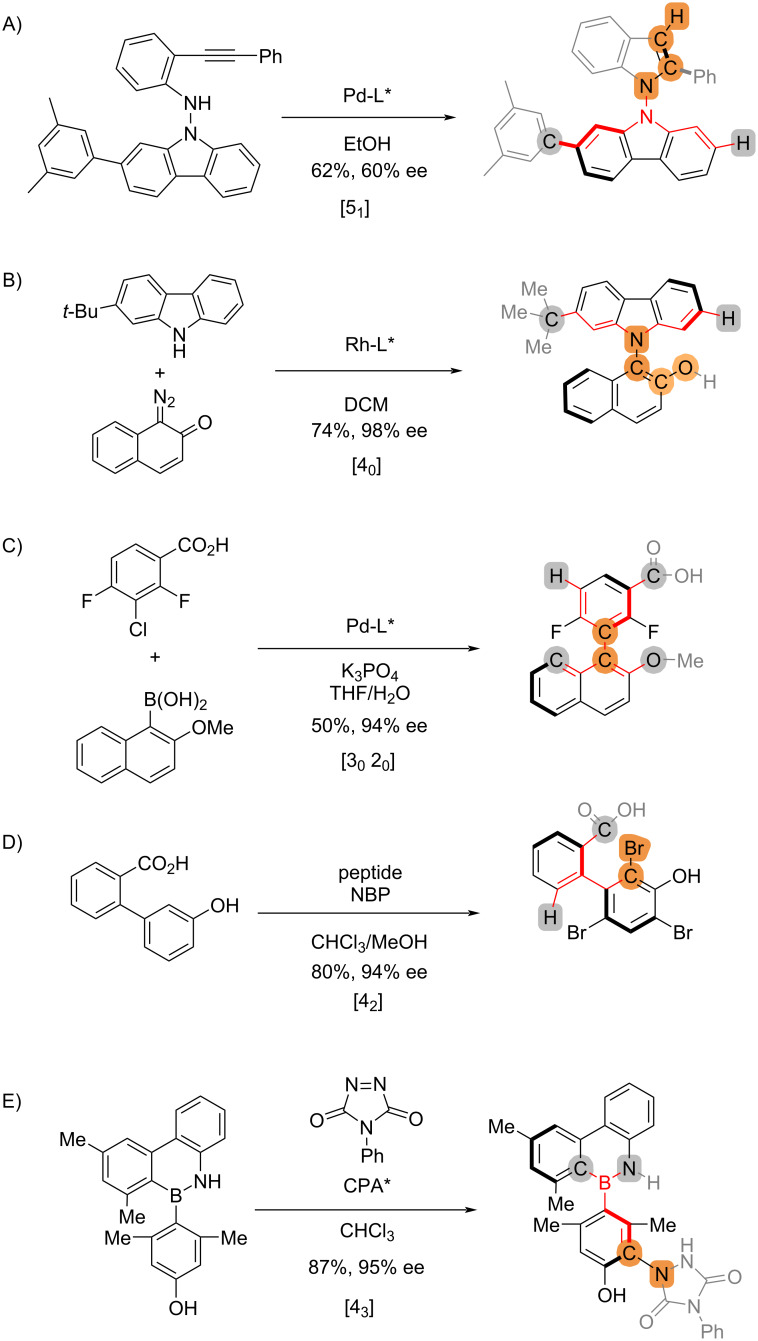
Examples of reactions that establish axial chirality derived from biaryls.

Different stereocontrol strategies could be employed to achieve asymmetric synthesis of axially chiral biaryls (Scheme 3). The stereocontrol connectivity indices are assigned following the 3-step procedure for all types of strategies including cyclization, biaryl coupling, and desymmetrization, irrespective of the chemical identity of the newly established chiral axis including C–C [10–11] (Scheme 3C and 3D), C–N [12] (Scheme 3B), N–N [13] (Scheme 3A), and C–B [14] (Scheme 3E) bonds. In the case of an asymmetric cross-coupling reaction (Scheme 3C) [10], both sets of the substituents on individual aryl groups are considered because neither is involved in the bond formation/cleavage. Accordingly, the catalyst-controlled atroposelective Suzuki–Miyaura coupling of biaryls is designated as [3_0_ 2_0_].

**Scheme 4:**
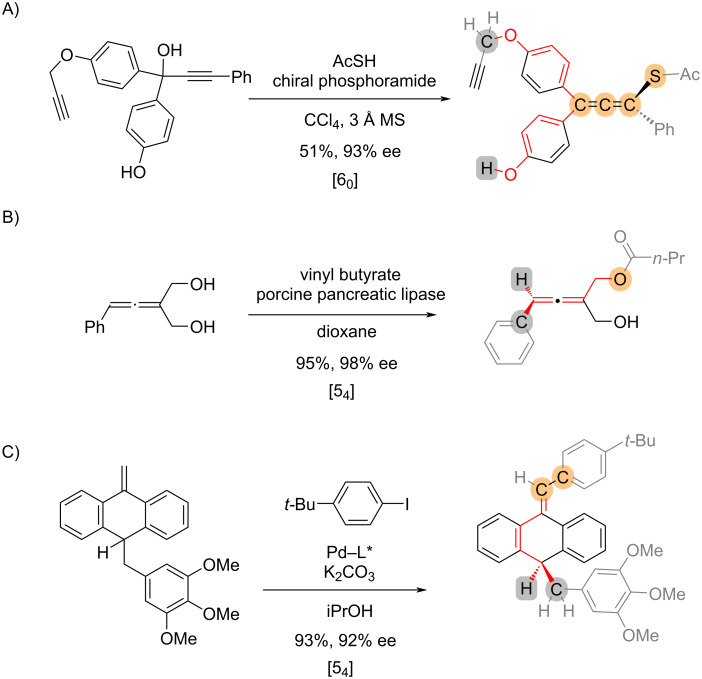
Examples of reactions that establish axial chirality derived from C=C bonds.

In addition to biaryls, axially chiral allenes are popular targets for asymmetric synthesis. Three examples of asymmetric reactions that form axially chiral allenes are shown in Scheme 4. For example, the enantioselective nucleophilic substitution to yield chiral allenes [15] is defined as [6_0_] (Scheme 4A), while the desymmetrizing reactions of allenes [16] (Scheme 4B) and anthracenylidene [17] (Scheme 4C) are both classified as [5_4_]. In the cases of Scheme 3A and 3C, the reaction intermediates (i.e., the *p*-quinone methide and the carbopalladation intermediate, respectively) are neglected to simplify the assignment, considering such information is inexplicit based on the chemical transformations alone and is not available in commonly used chemical databases. Therefore, the index is a denotation of the overall transformation, which is not always representative of the stereochemical-determining elementary step.

**Scheme 5:**
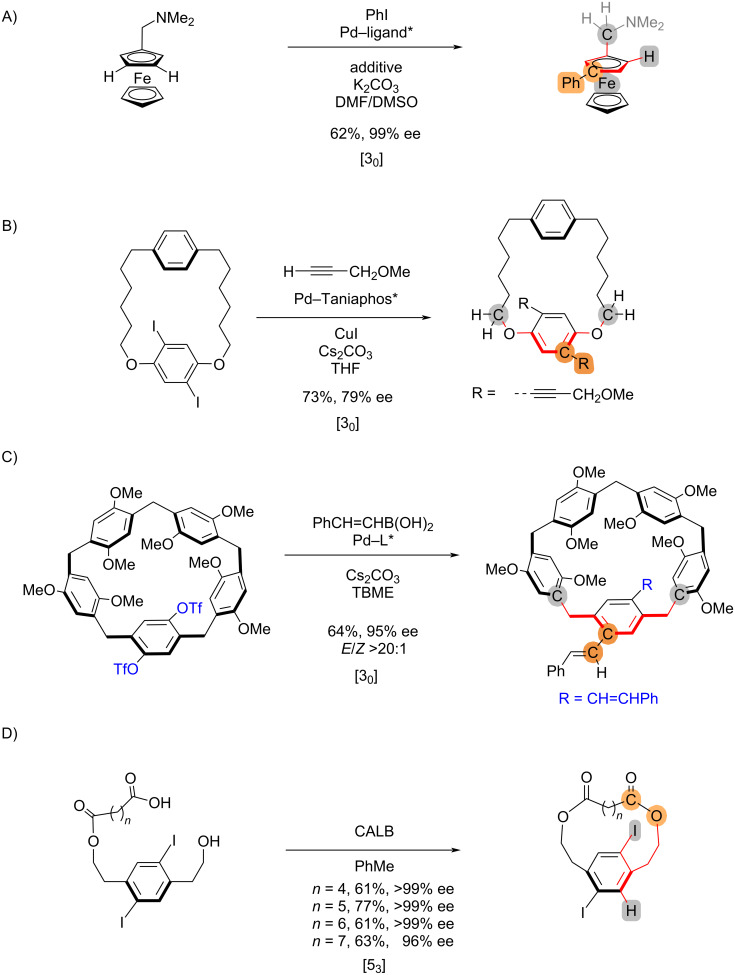
Examples of reactions that establish planar chirality.

The stereocontrol connectivity indices can be extended to reactions that establish planar chirality (Scheme 5). In the case of metallocenes, the metal is considered to be σ-bonded to the arene ring for convenience. In other words, the centroid atom is treated as a pseudo-tetrahedral center, with the metal regarded as one of the substituents. Accordingly, a C–H activation reaction that forms planar chiral ferrocenes [18] is assigned as [3_0_] (Scheme 5A). For cyclophanes, if the bond formation or cleavage is on the stereogenic arene, the stereochemical differentiation should at least be traced to the two pilot atoms that are directly attached, but not within the stereogenic plane – similar to the assignment of stereochemistry for cyclophanes. This way, the asymmetric Pd-catalyzed coupling [19–20] would be assigned as [3_0_] (Scheme 5B and 5C). On the other hand, if the bond formation/cleavage is within the macrocycle, the stereochemical differentiation atoms are traced to the stereogenic arene. Therefore, the CALB-mediated esterification to cyclophenes [21] is [5_3_], regardless of the length of the linkage (CH_2_)*_n_* (Scheme 5D).

**Scheme 6:**
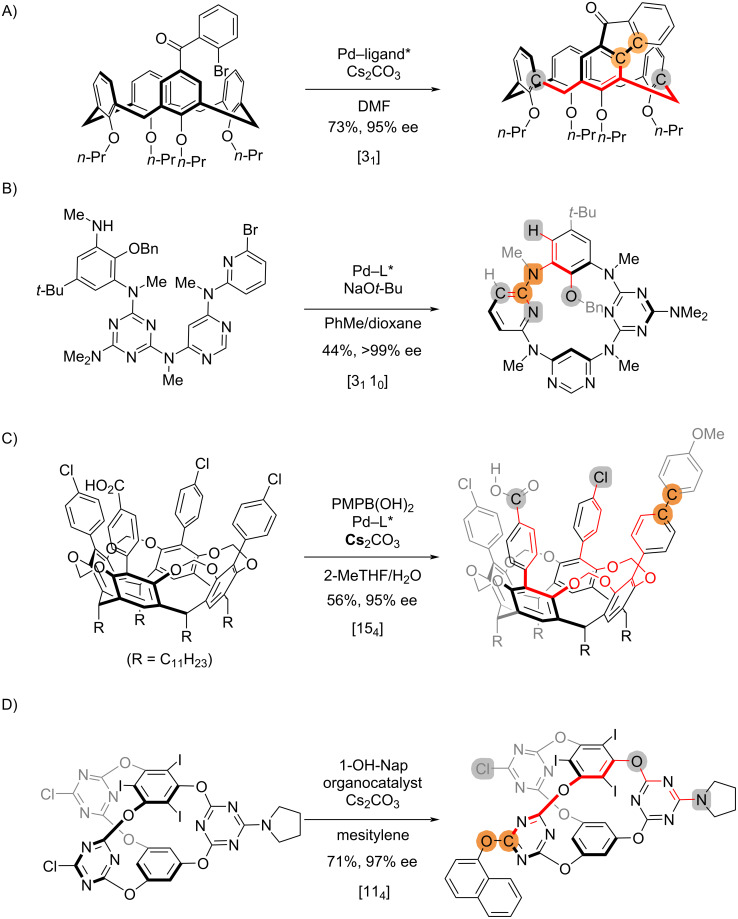
Examples of reactions that establish “inherent” chirality.

In recent years, asymmetric synthesis of “inherently chiral” macrocycles have gained growing attention [22]. Practically, such reactions that yield “inherently chiral” macrocycles can be treated similarly as planar chirality (Scheme 6). The synthesis of calix[4]arenes via C–H arylation [23] is [3_1_] (Scheme 6A), following the same procedure as in Scheme 5C. In analogy to Scheme 5B, the direct cyclization forging the planar chirality [24] in Scheme 6B is regarded as [3_1_ 1_0_], in which two stereogenic arenes are proximate and the two distal arenes are considered diastereomeric. The desymmetrization cross-coupling of cavitands [25] in Scheme 6C is designated as [15_4_]. In this case, the stereochemical differentiation viewed from the reaction sites can only be made beyond the two pilot atoms; the bond connectivity remains the same till the chloro group and the carboxyl group. Recently, Wang has reported an organocatalytic protocol to access inherently chiral cages via desymmetrization promoted by a chiral phase-transfer catalyst [26], which is designated as a [11_4_] process (Scheme 6D).

**Scheme 7:**
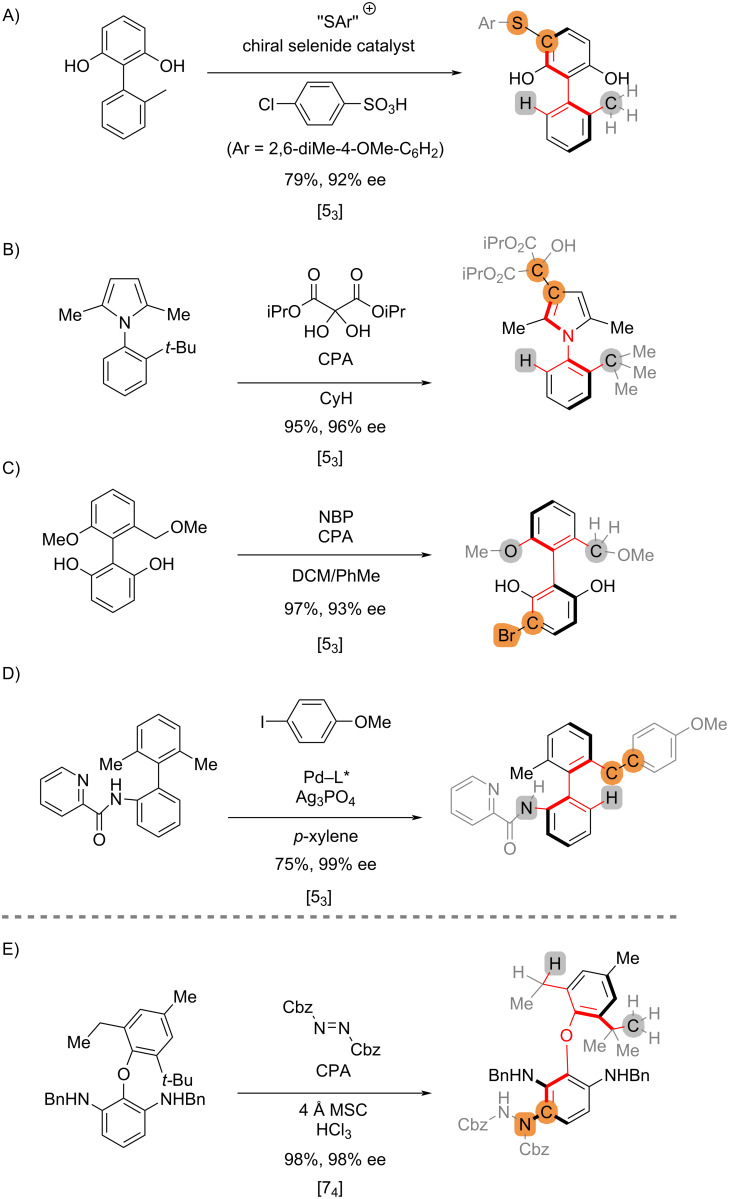
Parameterization of asymmetric reactions that establish axial chirality.

Asymmetric reactions can be categorized based on the stereocontrol connectivity index. For example, the remote desymmetrization reactions to axial chiralities [27–30] in Scheme 7A to 7D are all [5_3_] processes irrespective of the reaction types, the catalysts, and the scaffolds. The index corresponds to the minimal substructure of the prochiral substrates that a chiral catalyst needs to recognize, if the bond connectivity is the only factor considered, without taking into account the electronic, steric and conformational properties. For example, the stereocontrol of the desymmetrization reaction in Scheme 7E [31] would require the catalyst to discern between the two non-reactive *ortho*-substituents at distant points across the biphenyl ether backbone (H vs Me, highlighted in grey), which is reflected by the relatively large values of the index [7_4_].

Although we intend to avoid reaction intermediates in the assignment of the stereocontrol connectivity index, we recognize the limitations in cases that chemical bonds are cleaved and restored in the course of the transformations (e.g., Scheme 4A and 4C). Such limitations become obvious in the cases of multistep, multi-intermediate reactions [32–33], particularly in the case of helical chirality where chirality transfer of intermediates is common [34–39]. In addition, the 3-step procedure is not applicable to asymmetric synthesis of interlocked molecules including mechanically planar chiral rotaxanes and catenanes, where the bond connectivity between stereogenic components is absent [40–43].

Finally, we explored the automated process for the generation of indices following the 3-step procedure using a prototypic program coded using Python or through coaching GPT-4.1. Stereoselective reactions were input as SMILES (for Python) or InChI (for GPT-4.1) of the starting materials and products, and the corresponding indices were generated automatically. It is possible that functions could be expanded in the future so that the automated designation is applicable to various types of chirality and chemical structures.

**Figure 1:**
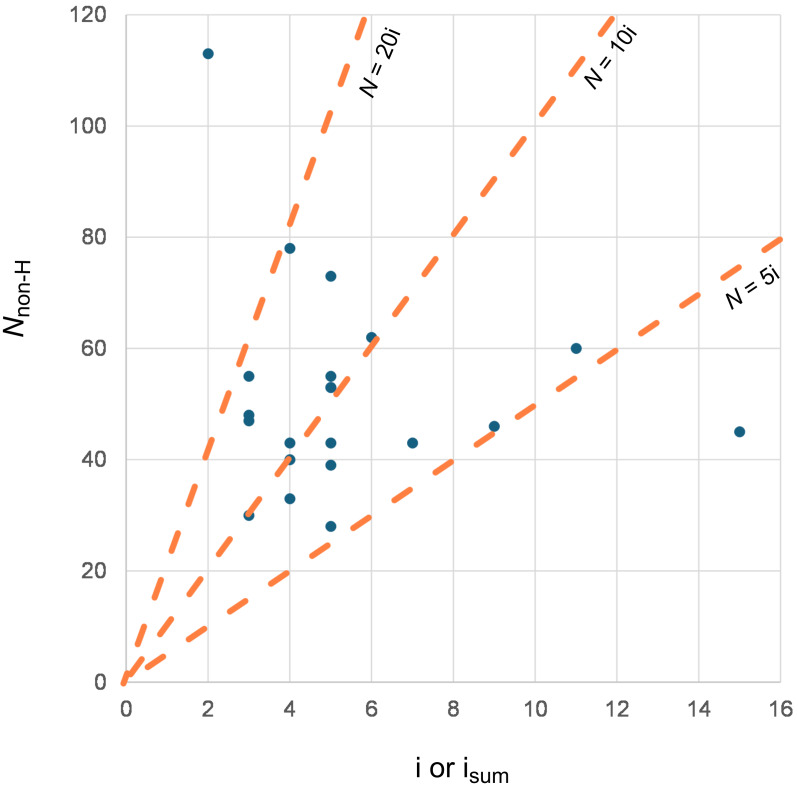
The relationship between the numbers of non-hydrogen atoms (*N*) in the chiral catalysts and the value of i in [i*_j_*].

The stereocontrol connectivity index is related to the minimum path across the prochiral substrate for effective transmission of chirality from the chiral catalyst to the product. In other words, the chiral catalyst needs to recognize, through electronic and steric effects, at least the structural features reflected by the stereocontrol connectivity index to induce enantioselectivity. Therefore, the stereocontrol connectivity index is relevant to the minimal dimension of an effective chiral catalyst for a given asymmetric transformation. We surveyed the relationship between the numbers of non-hydrogen atoms (*N*) in the chiral catalysts and the value of i (or sum of i) in [i*_j_*] for the 21 non-enzymatic transformations analyzed above (Figure 1). These transformations scattered across a broad area: 10 showed 20 × i > *N* >10 × i, and 9 showed 10 × i > *N* > 5 × i. Although neither *N*_non-H_ of the catalyst nor the index [i*_j_*] is related to the mechanism of stereocontrol, a low *N-*to-i ratio could occur when attractive secondary interactions between the catalyst and the substrates were involved in stereoinduction. In contrast, if this strategy is not applicable as a result of the non-polar nature of the substituents in the prochiral substrate, a high *N-*to-i ratio is expected to provide the necessary structural basis of the catalyst for stereoinduction by steric biasing.

The stereocontrol connectivity index does not reflect the difficulty of inducing stereoselectivity, which can be subjective. In particular, the actual spatial distances between critical structural elements cannot be measured simply by using bond connectivity. Nonetheless, the stereocontrol connectivity index can provide information on the stereochemical properties of reactions, beyond the existing binary (yes/no) stereochemistry classifications in widely used chemical databases such as CAS SciFinder and Reaxys. This exercise applies not only to existing asymmetric reactions but also to chemical transformations without effective stereocontrol, thus presenting new avenues for research.

## Conclusion

In summary, we have developed a stereocontrol connectivity index that parameterizes the stereogenic remoteness of asymmetric reactions. The index is applicable to chemical transformations that establish central chirality, axial chirality, planar chirality, and “inherent chirality”. The 3-step procedure for the designation of the stereocontrol connectivity index is derived from the chemical structures of the starting materials and the products, and it does not rely on knowledge in reaction mechanisms. Particularly, the application to reactions that establish non-central chirality allows a numeric measurement of the stereogenic remoteness – an intrinsic property of non-central chirality reflected by the large values of the indices (i ≥ 3). We anticipate that application of the stereocontrol connectivity index will facilitate the classification of stereoselective reactions and promote the development of challenging asymmetric transformations that establish non-central chirality.

## Supporting Information

File 1 The set of code (Python) and sample inputs and outputs, as well as the sample inputs and outputs using GPT-4.1 for the designation of stereocontrol connectivity indices are illustrated. The data used in Figure 1 is listed in Table S1.

## Data Availability

All data that supports the findings of this study is available in the published article and/or the supporting information of this article.
