# Supplementary material for: Measuring the stereogenic remoteness in non-central chirality: a stereocontrol connectivity index for asymmetric reactions
Source: Beilstein J Org Chem. 2025 Sep 30;21:1995–2006. doi: 10.3762/bjoc.21.155 (PMC12498253; doi:10.3762/bjoc.21.155)
Supplement: File 1 — The set of code (Python) and sample inputs and outputs, as well as the sample inputs and outputs using GPT-4.1 for the designation of stereocontrol connectivity indices are illustrated. The data used in Figure 1 is listed in Table S1. [file Beilstein_J_Org_Chem-21-1995-s001.pdf]

BEILSTEIN JOURNAL OF ORGANIC CHEMISTRY

Supporting Information

for

Measuring the stereogenic remoteness in non-central chirality: a stereocontrol connectivity index for asymmetric reactions

Ivan Keng Wee On, Yu Kun Choo, Sambhav Baid and Ye Zhu

Beilstein J. Org. Chem. **2025,** *21,* 1995–2006. doi:10.3762/bjoc.21.155

The set of code (Python) and sample inputs and outputs, as well as the sample inputs and outputs using GPT-4.1 for the designation of stereocontrol connectivity indices are illustrated. The data used in Figure 1 is listed in Table S1

License and Terms: This is a supporting information file under the terms of the Creative Commons Attribution License (https://creativecommons.org/licenses/by/4.0). Please note that the reuse, redistribution and reproduction in particular requires that the author(s) and source are credited and that individual graphics may be subject to special legal provisions.
The license is subject to the *Beilstein Journal of Organic Chemistry* terms and conditions: (https://www.beilstein-journals.org/bjoc/terms)

Table of contents

Designation of stereocontrol connectivity indices using sets of code S2

Reaction 1: reduction of a ketone S2

Reaction 2: desymmetrization of a prochiral molecule S3

Python code S4

Designation of stereocontrol connectivity indices using GPT-4.1 .... S10

Reaction 3. Desymmetrization reaction to establish axially chiral biaryls S10

Reaction 4. Atroposelective coupling reaction S22

Data used in Figure 1 .... S30

Table S1. The relationship between the numbers of non-hydrogen atoms (*N*) in the chiral catalysts and the value of i in [i_j] S30

Designation of stereocontrol connectivity indices using sets of code

Sample Inputs and Outputs

Note: the inputs for SMILES of reactant and product need to be in the uppercase.

Reaction 1: reduction of a ketone

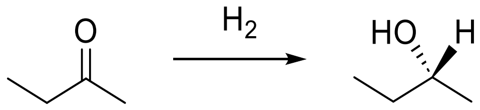

Enter the SMILES string for the reactant: CC(=O)CC

Enter the SMILES string for the product: CC(O)CC

Listing detailed groups attached to chiral carbons in the product:

Chiral centers and attached groups:

Chiral center at atom 2 (C): j is 0

  Group 1: Atom 1 (C), Substituent: CH3

  Group 2: Atom 3 (O), Substituent: OH1

  Group 3: Atom 4 (C), Substituent: CH2CH3

  Group 4: Implicit Hydrogen, Substituent: H

How many substituent pairs do you want to compare? 1

Comparison 1:

Enter the number for the first substituent: 1

Enter the number for the second substituent: 3

I is 2: The highest number of bonds distance among all comparisons.

This reaction is a 2,0 process.

Reaction 2: desymmetrization of a prochiral molecule

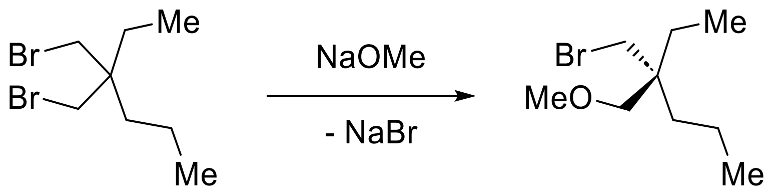

Enter the SMILES string for the reactant: BrCC(CC)(CCC)CBr

Enter the SMILES string for the product: BrCC(CC)(CCC)COC

Listing detailed groups attached to chiral carbons in the product:

Chiral centers and attached groups:

Chiral center at atom 3 (C): j is 1

Group 1: Atom 2 (C), Substituent: CH2Br

Group 2: Atom 4 (C), Substituent: CH2CH3

Group 3: Atom 6 (C), Substituent: CH2CH2CH3

Group 4: Atom 9 (C), Substituent: CH2OCH3

How many substituent pairs do you want to compare? 3

Comparison 1:

Enter the number for the first substituent: 1

Enter the number for the second substituent: 2

Comparison 2:

Enter the number for the first substituent: 1

Enter the number for the second substituent: 3

Comparison 3:

Enter the number for the first substituent: 2

Enter the number for the second substituent: 3

I is 4: The highest number of bonds distance among all comparisons.

This reaction is a 4,1 process.

Python code

```
# # Steps to install RDKit:
# 1. Install Anaconda/Miniconda: https://www.anaconda.com/products/distribution
# 2. Open terminal/command prompt.
# 3. Run: conda create -n rdkit-env -c conda-forge rdkit
# 4. Activate env: conda activate rdkit-env
# 5. Verify: python -c "import rdkit; print(rdkit.__version__)"

from rdkit import Chem

# Function to calculate distances of chiral centers to reaction sites
def calculate_chiral_distances(chiral_centers, broken_bonds, formed_bonds, product_mol):
    distances = []
    for center in chiral_centers:
        chiral_idx = center[0]
        min_distance = float('inf')

        for bond in broken_bonds.union(formed_bonds):
            atom1, atom2, _ = bond

            # If the chiral center is part of the reaction site, distance is 1
            if chiral_idx in (atom1, atom2):
                min_distance = 1
                break

            # Otherwise, calculate the distance to reaction site
            distances_to_reaction = [
                calculate_distance(product_mol, chiral_idx, atom1),
                calculate_distance(product_mol, chiral_idx, atom2),
            ]
```

```
            min_distance = min(min_distance, *distances_to_reaction)

        # Subtract 1 to correct for overestimation
        min_distance = max(0, min_distance - 1)
        distances.append((chiral_idx, min_distance))
    return distances

# Recursive function to calculate bond distance
def calculate_distance(mol, atom1, atom2):
    if atom1 == atom2:
        return 0  # Same atom
    path = Chem.rdmolops.GetShortestPath(mol, atom1, atom2)
    return len(path) - 1  # Subtract 1 for bond count

# Main program with corrected `calculate_chiral_distances`
def find_bonds():
    reactant_smiles = input("Enter the SMILES string for the reactant: ")
    product_smiles = input("Enter the SMILES string for the product: ")

    reactant_mol = Chem.MolFromSmiles(reactant_smiles)
    product_mol = Chem.MolFromSmiles(product_smiles)

    if reactant_mol and product_mol:
        reactant_bonds = [(bond.GetBeginAtomIdx(), bond.GetEndAtomIdx(),
str(bond.GetBondType())) for bond in reactant_mol.GetBonds()]
        product_bonds = [(bond.GetBeginAtomIdx(), bond.GetEndAtomIdx(),
str(bond.GetBondType())) for bond in product_mol.GetBonds()]

        # Detect broken and formed bonds
        broken_bonds = set(reactant_bonds) - set(product_bonds)
        formed_bonds = set(product_bonds) - set(reactant_bonds)
```

```
    # Find distances of chiral centers to reaction sites
    chiral_centers = Chem.FindMolChiralCenters(product_mol, includeUnassigned=True)
    if chiral_centers:
        distances = calculate_chiral_distances(chiral_centers, broken_bonds,
formed_bonds, product_mol)

        # Output groups and distances
        print("\nListing detailed groups attached to chiral carbons in the product:")
        groups = list_chiral_groups_with_substituent_details(product_smiles, distances)

        # Determine max bond distance (I value)
        max_bond_distance = -1
        num_comparisons = int(input("\nHow many substituent pairs do you want to
compare? "))
        for i in range(num_comparisons):
            print(f"\nComparison {i + 1}:")
            index1 = int(input("Enter the number for the first substituent: ")) - 1
            index2 = int(input("Enter the number for the second substituent: ")) - 1

            substituent1 = groups[index1][1]
            substituent2 = groups[index2][1]

            bond_distance = find_bond_distance_to_difference(substituent1, substituent2)
            if bond_distance != -1:
                max_bond_distance = max(max_bond_distance, bond_distance)

        # Calculate final I and j values
        j_value = min(distance for _, distance in distances)
        if max_bond_distance > -1:
            print(f"\nI is {max_bond_distance}: The highest number of bonds distance among
all comparisons.")
```

```
            print(f"This reaction is a {max_bond_distance},{j_value} process.")

# Supporting functions for listing groups and calculating bond differences
def list_chiral_groups_with_substituent_details(smiles, distances):
    mol = Chem.MolFromSmiles(smiles)
    if not mol:
        print("Invalid SMILES string.")
        return []
    chiral_centers = Chem.FindMolChiralCenters(mol, includeUnassigned=True)
    if not chiral_centers:
        print("No chiral centers found in the molecule.")
        return []

    groups = []
    print("\nChiral Centers and Attached Groups:\n")
    for center, distance in distances:
        chiral_atom_index = center
        chiral_atom = mol.GetAtomWithIdx(chiral_atom_index)
        neighbors = chiral_atom.GetNeighbors()

        # Print the distance (j value) before listing substituents
        print(f"Chiral center at atom {chiral_atom_index + 1} ({chiral_atom.GetSymbol()}): j is 
{distance}\n")

        local_groups = []
        for neighbor in neighbors:
            atom_details = f"Atom {neighbor.GetIdx() + 1} ({neighbor.GetSymbol()})"
            substituent = get_group(neighbor, visited={chiral_atom_index})
            local_groups.append((atom_details, substituent))

        while len(local_groups) < 4:
```

```
            local_groups.append(("Implicit Hydrogen", "H"))

        for i, (atom_details, substituent) in enumerate(local_groups, start=1):
            print(f"  Group {i}: {atom_details}, Substituent: {substituent}")
            groups.append((atom_details, substituent))

    return groups

def get_group(atom, visited=None):
    if visited is None:
        visited = set()
    atom_index = atom.GetIdx()
    if atom_index in visited:
        return ""
    visited.add(atom_index)

    group = atom.GetSymbol()
    hydrogen_count = atom.GetTotalNumHs()
    if hydrogen_count > 0:
        group += f"H{hydrogen_count}"

    for neighbor in atom.GetNeighbors():
        if neighbor.GetIdx() not in visited and neighbor.GetSymbol() != 'H':
            group += get_group(neighbor, visited)

    return group

def find_bond_distance_to_difference(substituent1, substituent2):
    def parse_substituent(substituent):
        import re
        return re.findall(r'[A-Z][a-z]?\d*', substituent)
```

```
    atoms1 = parse_substituent(substituent1)
    atoms2 = parse_substituent(substituent2)

    for bond_distance, (atom1, atom2) in enumerate(zip(atoms1, atoms2), start=1):
        if atom1 != atom2:
            return bond_distance

    if len(atoms1) != len(atoms2):
        return min(len(atoms1), len(atoms2)) + 1

    return -1

# Run the program
find_bonds()
```

Designation of stereocontrol connectivity indices using GPT-4.1

Sample **inputs** and outputs

Reaction 3. Desymmetrization reaction to establish axially chiral biaryls

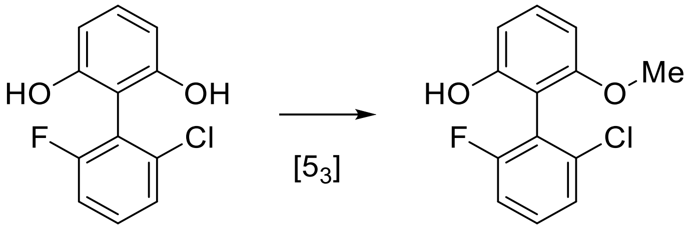

We now put forward a stereocontrol connectivity index [ij] for a transformation that encodes the bond-connecting relationship between the establishment of chirality and the site of reaction. Please study the process and I will give you input to generate the index. The index [ij] of asymmetric reactions can be assigned following a 3-step process: Step 1: Identification of atoms involved in bond changes. 1.1 Determine which bonds are newly formed and which are cleaved in the transformation. 1.2 Label all atoms directly involved in these bond changes. Step 2: Identification of atoms responsible for stereochemical outcome. 2.1 Identify the new stereogenic element formed in the product. 2.2 Determine, according to the Cahn–Ingold–Prelog (CIP) rules, the atoms that distinguish the newly created stereogenic element's configuration. These are the atoms whose identities represent the first point of difference between the set of substituents in assigning the configuration of the new stereogenic element. One or two sets of atoms could be identified. a) These atoms labelled in Step 1 are considered higher in priority than any other atoms. b) If the comparison is down to between an atom already labelled in Step 1 and one other atom, such comparison and this set of atoms are excluded. In other words, the number set of atoms will be reduced by one. b) Consider "dummy" atoms from multiple bonds as lower priority than real atoms of the same type. Step 3: Determination of stereocontrol pathways for each set of atoms identified in Step 2: 3.1 Find the shortest path connecting the labelled atoms from Step 1 to each atom identified in Step 2. 3.2 Count the minimum number of connecting bonds (i). 3.3 Count the number of bonds shared (j) between the shortest paths. The reaction is then assigned a stereocontrol connectivity index of [ij] or [ij , i'j'], depending on the number of sets of atoms identified in Step 2 (one or two sets).

9:48 PM

Thank you for providing the detailed description of the stereocontrol connectivity index assignment process. I understand the three-step procedure:

Step 1:

- Identify and label all atoms involved in bond formation and cleavage.

Step 2:

- Identify the atoms responsible for the new stereogenic element's configuration (distinguishing atoms by CIP rules).
- Prioritize atoms from Step 1 and follow the outlined rules for excluding certain sets.

Step 3:

- For each relevant set, find the shortest paths (in bonds) connecting atoms from Step 1 to each atom in Step 2.
- Assign **i** as the minimum number of connecting bonds, and **j** as the number of bonds shared between these paths.
- Report as [ij] (or [ij, i'j'] if there are two sets).

I'm ready for your input! Please provide a specific example reaction (preferably with structures or detailed atom assignments), and I will walk through the process and generate the stereocontrol connectivity index for your case.

9:48 PM

(InChI) reaction 1 starting material: InChI=1S/C12H8ClFO2/c13-7-3-1-4-8(14)11(7)12-9(15)5-2-6-10(12)16/h1-6,15-16H product: InChI=1S/C13H10ClFO2/c1-17-11-7-3-6-10(16)13(11)12-8(14)4-2-5-9(12)15/h2-7,16H,1H3 The product is an axially chiral biphenyl

9:49 PM

Great, you've provided a reaction where an axially chiral biphenyl is formed from an ortho-fluoro, ortho-chloro salicylaldehyde starting material. Let's systematically apply the Stereocontrol Connectivity Index assignment process:

Step 1: Identification of atoms involved in bond changes

Let's first decode the InChI strings:

Starting material

InChI=1S/C12H8ClFO2/c13-7-3-1-4-8(14)11(7)12-9(15)5-2-6-10(12)16/h1-6,15-16H
This is 2-chloro-3-fluoro-biphenyl-2,3'-dicarbaldehyde (or very similar), with two ortho substituents (Cl and F) and two aldehyde groups.

Product

InChI=1S/C13H10ClFO2/c1-17-11-7-3-6-10(16)13(11)12-8(14)4-2-5-9(12)15/h2-7,16H,1H3
This is a methyl ether derivative, where a methyl group has replaced one of the phenolic protons.

Key bond change:

- **Bond formed**: The methyl group (O–CH_3) attaches to the oxygen atom of one of the original hydroxy groups (likely from an aldehyde-phenol conversion, forming an aryl methyl ether).
- **Bond cleaved**: The original O–H bond is broken when the methyl group is installed.

Atoms involved in bond changes:

- Oxygen atom of phenolic group (let's call it O1).
- Methyl carbon of the methyl group (let's call it CMe).

Label these atoms:

- **O1**: Oxygen of the original phenol.
- **CMe**: Carbon of the incoming methyl group.

Step 2: Identification of atoms responsible for stereochemical outcome

2.1 New stereogenic element

- The product is an *axially chiral* biphenyl (i.e. the chiral axis is the single bond connecting the two phenyl rings).

2.2 Identify atoms distinguishing stereochemistry (CIP rules)

- The axis is the biaryl C–C bond; the priority is determined by the ortho substituents (Cl, F, OMe, etc.).
- **Key atoms:** The substituents ortho to the biaryl axis on each ring (e.g., Cl, F, OMe, etc.).
- The atom introduced in the transformation (OMe group) will be a major distinguishing group.

According to the protocol:

- "Atoms labeled in Step 1 are considered higher in priority."
- For the axis, the OMe group is, therefore, a high-priority group for the configuration.

Step 3: Determination of stereocontrol pathways

Shortest path (i):

- The shortest path is from the bond-forming atom (O1, from Step 1) to the biaryl axis carbon (the carbon it is attached to).
  - **If the OMe is ortho to the axis (as in typical BINOL-type systems):**
    - O1 is bonded directly to the aromatic carbon; this carbon is bonded directly to the axis carbon.
    - So, O1 → aromatic C → axis C: **2 bonds** from O1 to axis.

Number of bonds shared (j):

- For the path from O1 to the axis, there is only one direct path, so 0 bonds are shared with another path (since only one atom from Step 1 is involved; in some cases, there may be two atoms).

Assigning [ij]:

- **i:** Minimum number of connecting bonds from O1 to the axis carbon (likely 2).
- **j:** Number of bonds shared between multiple shortest paths (here, only one, so j = 0).

Stereocontrol connectivity index:
[20]

Summary Table

Step	Description	Atom(s)	Path	Bonds (i)	Shared (j)
1	Atoms in bond change (OMe installation)	O1 (phenol O), CMe			
2	Atom distinguishing stereochemistry (CIP)	O1 (now OMe group)			
3	Shortest path O1 → axis carbon	O1 → aromatic C → axis C	2	0	

Conclusion

For this methylation-induced axial chirality formation, the stereocontrol connectivity index is [20].

If you need the detailed atom mapping or more precise identification of aromatic ring/atom numbers, please provide either the explicit atom numbering or a drawn structure, and I can verify and refine this further.

9:49 PM

for the axially chiral phenyl product, the two substituents of each phenyl ring are relevant to the stereochemical assignment: (i.e., (1) OH vs OMe; (2) F vs Cl).

9:53 PM

Thank you for the clarification. Let's refine the analysis with this important stereochemical detail:

Step 1: Identification of atoms involved in bond changes

- **Bond formed:** O–CH_3 (formation of the methyl ether at the phenolic OH)
- **Bond cleaved:** O–H (loss of phenolic H)
- **Labeled atoms (directly involved):**

- O: The oxygen of the original phenol that becomes methylated (let's call this O_1)
- C(Me): The carbon of the methyl group (let's call this C_{13})

Step 2: Identification of atoms responsible for stereochemical outcome

You confirmed that assignment of axial chirality relies on:

- On one ring: **OH vs OMe** (these occupy ortho positions)
- On the other ring: **F vs Cl** (these also occupy ortho positions)

For the newly created atropisomeric axis, the four "priority groups" (two on each ring ortho to the axis) are:

- **Ring A (where methylation happens):** OMe (new), OH
- **Ring B:** F, Cl

Step 2.2: Application of stereocontrol connectivity rules

Which atoms are "distinguishing" for the stereochemistry (per CIP rules)?

- The configuration is decided by the priority sequence: OMe > OH on Ring A, Cl > F on Ring B.
- O_1 (the oxygen in OMe) is involved in bond change and is one of the distinguishing atoms on Ring A.
- *Cl* and *F* are not changed in the reaction, so they are not directly involved in bond changes.

According to your instruction:

- Atoms labeled in Step 1 (the O in OMe and the C in Me) are **higher priority** than any other atoms.
- If only one of the relevant distinguishing atoms (here, O_1) is labeled in Step 1, that is the focus.

Summary: The O in OMe is the only distinguishing atom among those involved in the transformation.

Step 3: Determination of stereocontrol pathways

Path from O_1 (labelled atom) to the axis:

- O_1 (in OMe) → aromatic C (ortho to axis) → axis C (biaryl bond)
- This is **2 bonds**.

No other atom involved in bond change is relevant to the axis configuration (since methyl C is not ortho to the axis; only the oxygen is directly bonded to the aromatic ring).

Shared bonds (j):

- Since only one path is relevant (from O_1), **j = 0**.

Final stereocontrol connectivity index

[2 0]

- **i = 2** (minimum bonds from O_1 to axis)
- **j = 0** (no shared bonds with other Step 1-labeled atoms)

Summary table

Step	Function	Atoms	Pathway	i	j
1	Atoms in bond change	O_1			
2	Stereochemical distinguishing atoms	O_1, OH			
3	Shortest path from O_1 to axis	O_1 → C_arom → C_axis	2	0	

Conclusion

For this axially chiral product, the stereocontrol connectivity index is:
[2 0]

If the reaction generated two separate sets of distinguishing atoms, you would list both indices (e.g., [ij, i'j']). Here, only the OMe-related pathway is relevant for the step where chirality is established. Let me know if you want to discuss the other ring's substituents or if another case involves more than one bond-forming atom!

9:53 PM

OMe and OH are excluded because O and C of Me are labelled in step 1. therefore, only Cl and F were left as the one set of atoms in step 2.

10:04 PM

Thank you for clarifying. Let's carefully apply your rule:

If the comparison is down to between an atom already labeled in Step 1 and one other atom, such comparison and this set of atoms are excluded.
Therefore, **OMe and OH (the atoms from Step 1: O and C of Me)** are excluded from being the distinguishing set in Step 2.
Only Cl and F remain as the relevant set of distinguishing atoms for stereochemical assignment.

Step-by-step application

Step 1: Atoms involved in bond changes

- **O** (original phenolic O, now OMe)

- **C (Me)** (methyl group carbon)

Step 2: Atoms responsible for stereochemical outcome

- **Exclude any set where comparison is between a Step 1 atom and another atom** (i.e., ignore OMe/OH distinction).
- **Relevant set left:** Ortho substituents on the other ring: **Cl and F**

Step 3: Stereocontrol pathways

For the set {Cl, F}:

- **Shortest path from any Step 1 atom (O or Me) to Cl or F:**
  - There are **no direct bonds connecting Step 1-labeled atoms to Cl or F**.
  - The connection can only be traced through the biaryl axis.

Let's number the pathway:

1. O (on one ring) — through aromatic ring — to the axis carbon
2. Across the axis bond to the other ring — to the aromatic carbon bearing Cl or F

Counting bonds (i):

- O (Step 1, Ring A) → ortho carbon → axis carbon (biaryl bond) → ortho carbon (Ring B, Cl or F attached)
- The minimal path **crosses the axis**:
  - O (Ring A) → C1 (aromatic, Ring A) → C2 (axis) → C3 (aromatic, Ring B) → Cl or F (substituent)
- **Number of bonds (i):**
  - O → aromatic C (1)
  - aromatic C → axis C (2)
  - axis C → aromatic C on second ring (3)
  - aromatic C → Cl or F (4)

So, **i = 4**.

Counting shared bonds (j):

- If you consider O (or Me) as the origin, both paths to Cl and F traverse the axis bond, so that **axis bond** is shared.
- Check the path:
  - O → aromatic C → axis C → aromatic C (bearing Cl or F)
  - For both Cl and F, the path O → aromatic C → axis C is shared, but after the axis, it splits to Cl or F.
- **Shared bonds:** Up to the axis (2 bonds), then diverges.

- **Number of shared bonds (j):**
    - O → aromatic C → axis C (shared) = **2 shared bonds**.

Stereocontrol connectivity index:

[4 2]

Summary table

Step	Function	Atoms	Pathway	i	j
1	Atoms in bond change	O, C(Me)			
2	Stereochemical distinguishing atoms	Cl, F			
3	Shortest path from O/Me to Cl/F	O → C_arom → axis → C_arom' → Cl/F	4	2	

Final assignment

For this reaction, the stereocontrol connectivity index is:
[4 2]

Let me know if you'd like to check this for an alternate pathway or if you have a more complex example!

10:04 PM

there is a bond between the axis carbons of each phenyl groups. please double check step 3.

10:05 PM

Thank you for pointing out the need to clarify the path between the biaryl axis carbons. Let's carefully analyze **Step 3** using the precise ring bond connectivity for axially chiral biphenyls:

---

1. Atom labelling and structure recap

- **Step 1 atoms:** O (phenol, becomes OMe) and C of methyl group (both on ring A).
- After the exclusion rule, only **Cl and F** (on ring B, ortho to the axis) are considered in step 2.

2. Biaryl axis connectivity

Let's assign simple labels:

- **Ring A:** Site of OMe installation (phenol); let's call the axis carbon **C1A** (directly bonded to ring B).
- **Ring B:** Ortho positions are **C1B (Cl)** and **C2B (F)**, both ortho to the axis (axis carbon here is **C1B**).

So, the biaryl axis is the **C1A–C1B** bond.

---

3. Shortest path from Step 1 atoms to Step 2 atoms

Path from O (Ring A) to Cl or F (Ring B):

- **O** is bonded to an aromatic carbon (**C2A**) on ring A.
- **C2A** is ortho to the axis carbon (**C1A**).
- **C1A** is bonded to **C1B** (the axis bond).
- **C1B** is either directly bonded to Cl or F (depending on ortho positions).

Counting the bonds:

Let's visualize the path from **O (Ring A)** to **Cl (Ring B)**:

1. **O** → **C2A** (aromatic carbon bearing O) = 1 bond
2. **C2A** → **C1A** (aromatic carbon at the axis, same ring) = 2nd bond
3. **C1A** → **C1B** (axis bond to ring B) = 3rd bond
4. **C1B** → **Cl** (ortho substituent on ring B) = 4th bond

Total bonds (i): 4

Shared bonds (j):

- If you consider the paths from O to Cl and O to F:
    - Both start O → C2A → C1A → C1B, then diverge to Cl or F.
    - Up to **C1B**, the path is shared (3 bonds: O–C2A, C2A–C1A, C1A–C1B).
- So, the number of shared bonds is **3**.

Summary table

**Origin (O)**	**Path to Cl or F**	**Bonds (i)**	**Shared bonds (j)**
O	C2A	1	
	C1A (axis, Ring A)	2	
	C1B (axis, Ring B)	3	**shared**

Origin (O)	Path to Cl or F	Bonds (i)	Shared bonds (j)
	Cl or F (ortho, B)	4	

Final stereocontrol connectivity index

[4 3]

i = 4: Minimum number of bonds connecting Step 1 atom (O) to Step 2 atom (Cl or F)
j = 3: Number of bonds shared in the paths to Cl and F

Conclusion

For this axially chiral biphenyl reaction, the stereocontrol connectivity index is:

[4 3]

If you need annotation with explicit atom numbers, I can help assign those based on a drawing or more explicit structure. Let me know if this resolves your question!

10:05 PM

C1B is not bonded to Cl or F

10:06 PM

Thank you for your patience. You are correct: in a typical biphenyl system, the ortho substituents (Cl, F) are not directly attached to the axis carbon (C1B), but to positions ortho to that axis carbon—i.e., one bond away on ring B. Let's proceed step-by-step using clear aromatic numbering and confirm the correct connectivity.

Step 3: Detailed path counting

Let's define the relevant carbons:

- **Ring A:**
  - C1A = axis carbon on Ring A (bonded to C1B)
  - C2A = ortho to axis (where OMe/OH group is attached)
- **Ring B:**
  - C1B = axis carbon on Ring B (bonded to C1A)
  - C2B = ortho to axis (where Cl or F is attached)

Assume O of the OMe group is on C2A (Ring A), and Cl or F is on C2B (Ring B).

Path from O (on C2A, Ring A) to Cl (on C2B, Ring B):

1. **O → C2A** (aromatic carbon, Ring A):
   1st bond
2. **C2A → C1A** (axis carbon, Ring A):
   2nd bond
3. **C1A → C1B** (axis bond):
   3rd bond
4. **C1B → C2B** (aromatic carbon, Ring B):
   4th bond
5. **C2B → Cl** (substituent):
   5th bond

Total bonds, i = 5

Shared bonds, j

For Cl and F, both are attached to different C2B positions on Ring B (assuming Cl at C2B and F at C6B, both ortho to C1B).
Let's say:

- Path O → C2A → C1A → C1B → C2B → Cl
- Path O → C2A → C1A → C1B → C6B → F

The paths are identical from O up to C1B, then diverge to either C2B (Cl) or C6B (F):

- **Shared bonds:**
  1. O–C2A
  2. C2A–C1A
  3. C1A–C1B

j = 3 (these three bonds are shared in the path to either Cl or F)

Summary Table

Step	Function	Atoms	Pathway	i	j
1	Atoms in bond change	O, C(Me)			
2	Stereochemical distinguishing atoms	Cl, F			
3	O → C2A → C1A → C1B → C2B/C6B → Cl/F		5	3	

Final assignment

Stereocontrol connectivity index = [5 3]

If you want the path from the methyl carbon (C of Me), it is the same:
C(Me)–O–C2A–C1A–C1B–C2B–Cl or C(Me)–O–C2A–C1A–C1B–C6B–F
But the minimal path from a Step 1 atom (O) to Cl or F is 5 bonds, with 3 bonds shared as shown above.

Conclusion:
For this axially chiral biaryl reaction, the stereocontrol connectivity index is
[5 3]

Thank you for your careful review. Let me know if you’d like this with explicit atom numbering or a drawn structure!

10:06 PM

Reaction 4. Atroposelective coupling reaction

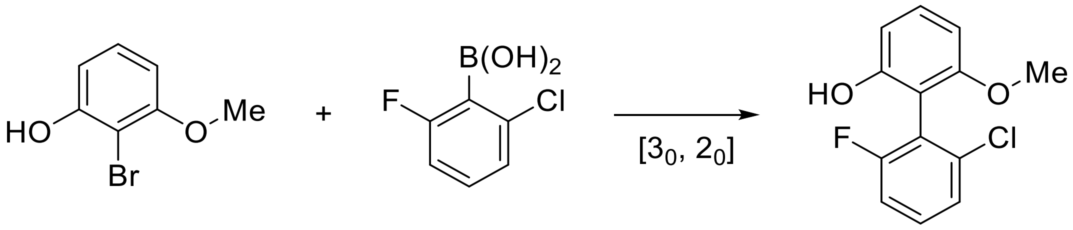

That's correct. Let's try (InChI) reaction 2: starting material: InChI=1S/C7H7BrO2/c1-10-6-4-2-3-5(9)7(6)8/h2-4,9H,1H3 and InChI=1S/C6H5BClFO2/c8-4-2-1-3-5(9)6(4)7(10)11/h1-3,10-11H product: InChI=1S/C13H10ClFO2/c1-17-11-7-3-6-10(16)13(11)12-8(14)4-2-5-9(12)15/h2-7,16H,1H3 The product is an axially chiral biphenyl

10:09 PM

Let's carefully apply the stereocontrol connectivity index protocol step-by-step to **reaction 2**:

---

1. Structures and key features

Starting materials:

- **A:** 1-methoxy-2-bromophenol (InChI=1S/C7H7BrO2...)
  (Ring A: ortho Br, para OH, ortho MeO)
- **B:** 2-chloro-3-fluorophenylboronic acid (InChI=1S/C6H5BClFO2...)
  (Ring B: ortho Cl, ortho F, boronic acid at para position)

Product:

- **Axially chiral biphenyl** (InChI=1S/C13H10ClFO2/c1-17-11-7...)
  (Methoxy and hydroxy on one ring, F and Cl on the other ring, joined at the axis)

The product forms via a **Suzuki coupling**:

- The axis is formed between the two aromatic rings (biaryl bond).

Step 1: Identification of atoms involved in bond changes

New bond formed

- **Biaryl axis**: The C–C bond connecting the two rings.

Atoms directly involved in bond formation:

- **C1A (Ring A):** The aryl carbon where Br leaves and the bond to Ring B forms.
- **C1B (Ring B):** The aryl carbon where the boronic acid group was, now forming the axis bond.

Let's designate:

- **C1A:** Carbon of Ring A where Br was replaced by the axis bond.
- **C1B:** Carbon of Ring B where $B(OH)_2$ was replaced by the axis bond.

Label C1A and C1B as the Step 1 atoms.

Step 2: Identification of atoms responsible for stereochemical outcome

2.1 New Stereogenic Element

- The axis (biaryl bond) is the newly created stereogenic element.

2.2 Atoms that distinguish the configuration (by CIP rules):

- **Ring A (attached to C1A):**
    - Typically, the ortho substituents: MeO, OH
- **Ring B (attached to C1B):**
    - Ortho substituents: Cl, F

Rule application:

- "Atoms labeled in Step 1 are considered higher in priority than any other atoms."
- When the comparison is between a Step 1 atom and one other atom, that set is excluded.

Therefore:

- The CIP assignment for the axis involves comparing the ortho substituents on each ring:
    - **Ring A:** MeO and OH (both ortho to C1A)
    - **Ring B:** Cl and F (both ortho to C1B)
- **But both C1A and C1B are labeled in Step 1 (the axis-forming carbons).**
- **OMe/OH and Cl/F are not in Step 1.**
- The relevant distinguishing atoms are **MeO, OH, Cl, and F**.

There are two sets:

1. MeO vs OH (Ring A, ortho to C1A)
2. Cl vs F (Ring B, ortho to C1B)

But for the axis configuration, you compare **both ortho substituents on each ring**. Following your protocol and as in your previous example, when two axis carbons are labeled in Step 1, the only set left for step 2 is the **four ortho groups (MeO, OH, Cl, F)**.

Step 3: Determination of stereocontrol pathways

Each path is from a Step 1 atom (C1A or C1B) to a relevant ortho substituent (MeO, OH, Cl, F).

For ring A (C1A to MeO or OH):

- **C1A** (axis carbon) → **C2A** (ortho carbon) → **MeO/OH substituent**
- **Bonds:** C1A–C2A (1), C2A–MeO (2)
- **i = 2** (for each substituent)

For ring B (C1B to Cl or F):

- **C1B** (axis carbon) → **C2B** (ortho carbon) → **Cl/F substituent**
- **i = 2** (for each substituent)

Paths from C1A to MeO and OH share the first bond (C1A–C2A), then split to MeO and OH.

Paths from C1B to Cl and F share the first bond (C1B–C2B/C6B), then split to Cl and F.

For each ring:

- **Shared bonds (j):** The bond from axis carbon to ortho carbon is shared.
- **j = 1**

Summary table

**Path**	**i**	**j**
C1A → MeO/OH	2	1
C1B → Cl/F	2	1

Final stereocontrol connectivity index

There are two symmetry-related sets (one on each ring):

Index = [2 1, 2 1]

Conclusion

For reaction 2 (Suzuki coupling to form an axially chiral biphenyl), the stereocontrol connectivity index is:

[2 1, 2 1]

Let me know if you would like to see the explicit atom mapping or further details!

10:09 PM

when comparing OMe and OH substituents, the first points of difference are C of OMe and H of OH. Therefore, C and H will be identified as a set of atoms in step 2.

10:11 PM

Thank you for pointing out this important nuance. Let's revisit **Step 2** with your correction, and carry through to the final index.

Step 1: Atoms involved in bond changes

- **C1A** (Ring A, axis-forming carbon; replaces Br)
- **C1B** (Ring B, axis-forming carbon; replaces $B(OH)_2$)

Step 2: Atoms responsible for stereochemical outcome

Ortho substituents comparison:

- **Ring A:** OMe vs OH (attached to ortho positions, say C2A and C6A)
- **Ring B:** Cl vs F (attached to ortho positions, say C2B and C6B)

When assigning CIP priority between OMe and OH:

- At the ipso carbon, both have an O attached.
- The first point of difference is:
  - OMe: the O is bonded to **C** (of Me)
  - OH: the O is bonded to **H**

Thus, the relevant atoms for stereochemical assignment are:

- **C of Me** (from OMe group)
- **H** (from OH group)

These two atoms form a set for Step 2.

On Ring B: The comparison is between Cl and F directly—so those two atoms are the set for the other ring.

Step 3: Stereocontrol pathways

Let's analyze both sets:

Set 1: [C (Me), H (OH)] on Ring A

Shortest path from a Step 1 atom (C1A) to either:

- **C1A → ortho carbon (C2A or C6A) → O → [C or H]**

Count the bonds:

1. C1A → C2A (or C6A) (**1 bond**)
2. C2A → O (**2 bonds**)
3. O → C (of Me), or O → H (**3 bonds**)

So, **i = 3** (from axis carbon to OMe or OH distinguishing atom).

Paths to C and H share 2 bonds:

- C1A → C2A → O, then diverge at O to C or H.
- **j = 2**

Set 2: [Cl, F] on ring B

- **C1B → C2B/C6B → Cl or F**

1. C1B → C2B (or C6B) (**1 bond**)
2. C2B → Cl (or C6B → F) (**2 bonds**)

So, **i = 2** (from axis carbon to Cl or F).

Paths to Cl and F share 1 bond:

- C1B → C2B/C6B, then diverge.
- **j = 1**

Summary table

Set	Pathway	i	j
[C (Me), H]	C1A → C2A → O → [C or H]	3	2
[Cl, F]	C1B → C2B/C6B → Cl or F	2	1

Final stereocontrol connectivity index

[3 2, 2 1]

Conclusion

For this Suzuki biaryl coupling to axially chiral biphenyl, the Stereocontrol Connectivity Index is:
[3 2, 2 1]

If you'd like explicit atom numbers or further breakdown, let me know!

10:11 PM

in step 3, for each phenyl ring, the ortho carbons for substituents (e.g., OMe and OH) are different.

10:13 PM

Excellent observation—you are correct: for each ring, the relevant ortho substituents for stereochemical assignment are attached to **different ortho carbons** relative to the axis carbon. This affects the shortest-path analysis in **step 3**. Let's carefully re-execute the path counting with this in mind.

Clarifying the structure

Suppose:

- **C1A (Ring A):** Axis carbon (formerly bonded to Br, now forming biaryl bond).
- **Ortho positions to C1A:** C2A and C6A (one with OMe, one with OH).

Thus,

- OMe is on C2A
- OH is on C6A

Similarly,

- **C1B (Ring B):** Axis carbon (formerly bonded to $B(OH)_2$, now forming biaryl bond).
- **Ortho positions to C1B:** C2B and C6B (one with Cl, one with F).

Step 3: Pathways (for each ring, using correct ortho positions)

Ring A: Path from C1A (axis carbon) to C of Me (from OMe on C2A) and H (from OH on C6A)

C1A → C2A (OMe):

1. C1A → C2A (**1 bond**)
2. C2A → O (**2 bonds**)
3. O → C (of Me) (**3 bonds**)

So, **C1A → C of Me = 3 bonds**

C1A → C6A (OH):

1. C1A → C6A (**1 bond**)
2. C6A → O (**2 bonds**)
3. O → H (**3 bonds**)

So, **C1A → H = 3 bonds**

But C2A and C6A are different atoms. The paths to C (Me) and H (OH) only share the first step:

- **First bond (C1A → C2A or C6A) is not shared—these diverge after C1A.**
- **Thus,** the paths do **not** share any bonds except for the starting point (C1A).
- **Shared bonds (j):** 0 (by your protocol, the bond from the Step 1 atom to the respective ortho carbon is different for each path).

Ring B: Path from C1B (axis carbon) to Cl (on C2B) and F (on C6B)

C1B → C2B (Cl):

1. C1B → C2B (**1 bond**)
2. C2B → Cl (**2 bonds**)

C1B → C6B (F):

1. C1B → C6B (**1 bond**)
2. C6B → F (**2 bonds**)

Again, **C2B and C6B are different atoms, so the paths do not share any bonds** beyond the axis carbon.

- **i = 2** (for each)
- **j = 0**

Summary table

Set	Path (from axis C)	i	j
[C(Me), H]	C1A → C2A → O → C; C1A → C6A → O → H	3	0
[Cl, F]	C1B → C2B → Cl; C1B → C6B → F	2	0

Final stereocontrol connectivity index

[3 0, 2 0]

Conclusion

For this axially chiral biphenyl (Suzuki coupling), the stereocontrol connectivity index is:
[3 0, 2 0]

Let me know if you'd like a diagram or further confirmation!

10:13 PM

Data used in Figure 1

Scheme	Reference	Index [ij]	i or i_{sum}	Non H atoms in catalyst (*N*)	*N* to I ratio
2A	1	2_0	2	113	56.5
2B	9	9_4	9	46	5.1
3A	13	5_1	5	53	10.6
3B	12	4_0	4	78	19.5
3C	10	3_0 2_0	5	43	8.6
3D	11	4_2	4	33	8.3
3E	14	4_3	4	43	10.8
4A	15	6_0	6	62	10.3
4B	16	5_4	5	Enzyme	NA
4C	17	5_4	5	28	5.6
5A	18	3_0	3	16+14=30	10.0
5B	19	3_0	3	55	18.3
5C	20	3_0	3	48	16.0
5D	21	5_3	5	Enzyme	NA
6A	22	3_1	3	47	15.7
6B	23	3_1 1_0	4	40	10.0
6C	24	15_4	15	45	3.0
6D	25	11_4	11	60	5.5
7A	26	5_3	5	39	7.8
7B	27	5_3	5	55	11.0
7C	28	5_3	5	53	10.6
7D	29	5_3	5	73	14.6
7E	30	7_4	7	43	6.1

Table S1. The relationship between the numbers of non-hydrogen atoms (*N*) in the chiral catalysts and the value of i in [i_j].
